# Thermal adaptability of *Kluyveromyces marxianus* in recombinant protein production

**DOI:** 10.1186/1475-2859-12-34

**Published:** 2013-04-15

**Authors:** Stefano Raimondi, Elena Zanni, Alberto Amaretti, Claudio Palleschi, Daniela Uccelletti, Maddalena Rossi

**Affiliations:** 1 Department of Life Sciences, University of Modena and Reggio Emilia, Via Campi 183, Modena, 41125, Italy; 2 Department of Biology and Biotechnology Charles Darwin, University of Rome “La Sapienza”, Piazzale Aldo Moro 5, Rome, 00185, Italy

## Abstract

**Background:**

*Kluyveromyces marxianus* combines the ease of genetic manipulation and fermentation with the ability to efficiently secrete high molecular weight proteins, performing eukaryotic post-translational modifications. It is able to grow efficiently in a wide range of temperatures. The secretion performances were analyzed in the host *K. marxianus* L3 in the range between 5°C and 40°C by means of 3 different reporter proteins, since temperature appears a key parameter for production and secretion of recombinant proteins.

**Results:**

The recombinant strains were able to grow up to 40°C and, along the tested temperature interval (5-40°C), the specific growth rates (μ) were generally lower as compared to those of the untransformed strain. Biomass yields were slightly affected by temperature, with the highest values reached at 15°C and 30°C. The secretion of the endogenous β-fructofuranosidase, used as an internal control, was efficient in the range of the tested temperature, as evaluated by assaying the enzyme activity in the culture supernatants. The endogenous β-fructofuranosidase production was temperature dependent, with the highest yield at 30°C. The heterologous proteins HSA, GAA and Sod1p were all successfully produced and secreted between 5°C and 40°C, albeit each one presented a different optimal production temperature (15, 40, 5-30°C for HSA, GAA and Sod1p, respectively).

**Conclusions:**

*K. marxianus* L3 has been identified as a promising and flexible cell factory. In a sole host, the optimization of growth temperatures for the efficient secretion of each individual protein can be carried out over a wide range of temperatures.

## Background

*Kluyveromyces marxianus* is one of the alternative yeasts for which an efficient transformation system was developed [1]. It has a long history of usage in food fermentation, and holds the GRAS status (Generally Regarded As Safe) that allows straightforward food and pharmaceutical applications of this microorganism and its derivatives [2]. *K. marxianus* grows on cheap lactose based media, such as whey and inulin, ferments xylose to ethanol, and does not require expensive explosion-proof plants which are necessary for methylotrophic yeasts [3,4].

Ethanol production represents one of the pivotal field of *K. marxianus* utilization. In the fermentation of renewable biomass residues, its capability to simultaneously consume glucose and xylose can be exploited in order to maximize ethanol productivity [4]. Recently, *K. marxianus* was also employed for tequila production at industrial level: the strain showed a higher osmotic (22% w/v) and ethanol (10% v/v) tolerance, producing high levels of volatiles [5].

However, the intrinsic advantages of *K. marxianus* over other yeasts will be better utilized for a variety of biotechnological applications as more and well-defined genetic engineering tools will become available. Recently, four *S. cerevisiae* promoters were characterized in *K. marxianus* for promoter strength, time-dependent changes, and stochastic gene expression pattern [6]. In addition, the genome of the thermotolerant *K. marxianus* KCTC 17555 that can convert inulin-rich plant biomass into ethanol and/or platform biochemicals was sequenced and analysed [7].

For heterologous gene expression, *K. marxianus* represents a valid alternative because of its outstanding secretory capabilities [8-10]. *K. marxianus* can also produce eukaryotic proteins since it modifies foreign proteins according to a general eukaryotic scheme. Moreover, *K. marxianus* has been exploited as a cell factory to obtain valuable enzymes, showing retention of activity in a large temperature interval [11]*.*

A peculiar trait of this yeast is the capacity to grow within a wide interval of temperatures, ranging from 4°C to 45°C. This feature has been investigated in a study where the adaptive responses at different temperatures of psychrophilic and mesophilic yeasts were compared [12]. The main objective of the current study was to explore the capability of *K. marxianus* to secrete various heterologous proteins at different temperatures. In fact, for both eukaryotic and prokaryotic hosts, it has been reported that growth temperature affects the yield of properly folded recombinant proteins [13,14]. In *E. coli* a common strategy to express the target protein in a soluble state consists in evaluating different growth temperatures. Temperature lowering improves the solubility of the proteins by diminishing aggregation as a result of a decrease in the production rate, allowing the newly synthesized chain to fold properly [15,16]. Several studies explored the effect of the growth temperature on the expression and secretion of heterologous proteins also in yeasts, albeit, in most cases, the authors investigated limited arrays of temperatures, generally ranging between 15 and 30°C [17-19].

Herein, the impact of different temperatures on endogenous and recombinant protein production and secretion was assessed in *K. marxianus* within a wider range of temperatures, ranging between 5 and 40°C. The reporter genes encoding the glucoamylase (GAA) from the yeast *Arxula adeninivorans*[20], the recombinant human serum albumin (HSA) [21], and the *Kluyveromyces lactis* Cu^2+^/Zn^2+^ superoxide dismutase (SOD1) [22] were used.

## Results and discussion

To the best of our knowledge, the capability to grow both at mesophilic and psychrophilic temperatures has never been investigated for a unique eukaryotic host of recombinant proteins. *Kluyveromyces marxianus* can grow in a wide range of temperatures, it is still an underexploited biotechnological system, and few studies have so far explored its potential for heterologous protein production [8,9]. In the present work, *K. marxianus* has been challenged as a cell factory model to investigate the impact of temperature on the secretion efficiency of endogenous and recombinant proteins.

### Specific growth rate and biomass yield of *Kluyveromyces marxianus* L3

Growth rate and biomass yield are physiological features of major importance for a “cell factory” organism in order to reach high volumetric productivities of recombinant proteins. It is generally assumed that growth at temperatures below the optimal value positively affects production and secretion of recombinant proteins. In this perspective, we investigated *K. marxianus* L3 specific growth rates (μ) and biomass yield (g L^-1^) at different temperatures (5, 15, 30, 40, and 45°C; Table 1), exploiting the advantage of this heat-tolerant yeast, which optimum temperature for growth falls generally around 42°C.

**Table 1:** **Effects of the temperature on growth of *K. marxianus *L3 wild-type and recombinant strains**

**T(°C)**	**Wild-type**	**Wild-type**	**Wild-type**	**rHSA**	**GAA**	**KlSod1p**
	**DW (gL**^**-1**^**)**	**Q**_**x**_**(gL**^**-1**^ **h**^**-1**^**)**	**μ (h**^**-1**^**)**	**μ (h**^**-1**^**)**	**μ (h**^**-1**^**)**	**μ (h**^**-1**^**)**
5	10.5^#^	0.096	0.041	0.034^a^	0.031^a^	0.034^a^
15	12.8^‡^	0.355	0.171	0.138^a^	0.160	0.144^a^
30	12.9^‡^	0.479	0.377	0.321^a^	0.341^a^	0.271
40	10.6^#^	0.443^‡^	0.439	0.293^a^	0.369	0.313^a^
45	9.8	0.426^‡^	0.464	-	-	-

The strains were cultured at different temperatures in YPD medium containing 20 g L^-1^ glucose. The specific growth rates (μ), the biomass yield (expressed as dry weight grams *per* litre of culture) and the biomass volumetric productivities (Q_X_) are reported as a function of growth temperature. The values are means of three independent experiments, with standard deviations always lower than 5%. Superscripts within a column (symbols) and within a row (letters) indicate statistically similar means, P > 0.05.

As expected, the temperature markedly affected the specific growth rate (μ). The μ increased if the temperature raised, and it was highest at 45°C (0.464 h^-1^). This behavior was in accordance with data reported in literature indicating that all the *K. marxianus* strains are able to grow at 42°C, and only few strains can grow up to 48°C [23].

Biomass yields were slightly affected by temperature, with the highest values reached at 15 and 30°C (12.8 and 12.9 g L^-1^, respectively). These high yields confirmed the excellent capability of *K. marxianus* to convert substrate into biomass [24]. The lower values at the highest temperatures (10.6 and 9.8 g L^-1^ at 40 and 45°C, respectively) suggested that, even if the growth rate was high, cell reproduction at the highest temperature required a metabolic cost that affected the biomass yield.

### Secretion of endogenous β-fructofuranosidase

**Figure 1:**
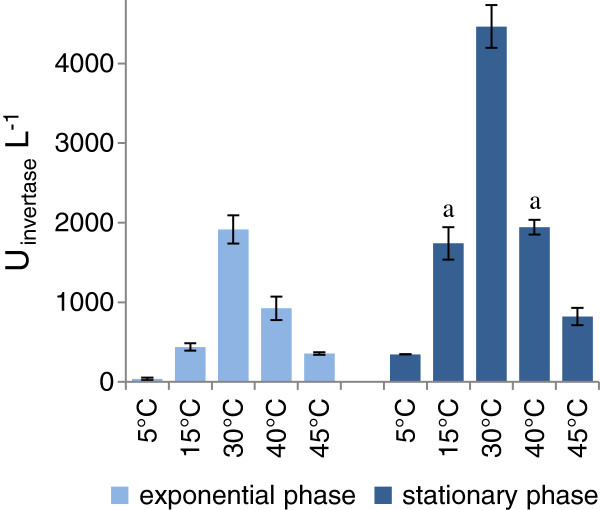
**Effects of the growth temperature on secretion of the β-fructofuranosidase.** β-fructofuranosidase has been measured as inulinase activity at 5 - 40°C, both at the growth and stationary phases. The values are means of three independent experiments ± standard deviations. Superscripts indicate statistically similar means (P > 0.05). Letters are used to compare samples collected at the same growth phase, grown at different temperatures. Symbols are used to compare samples grown at the same temperature, collected at diverse growth phases (exponential or stationary).

In order to assess the secretory capability of the host strain *K. marxianus* L3, the activity of endogenous β-fructofuranosidase (EC 3.2.1.26) was analyzed in the supernatant of cultures grown between 5 and 45°C. At all the tested temperatures, enzyme activity increased during both the growth and the stationary phases (Figure 1). The extracellular β-fructofuranosidase activity was temperature dependent, with the highest yield at 30°C (Figure 1). With the exception of 5°C, the enzyme production was abundant also at lower and higher temperatures. This result was consistent with the data reported in literature [25-27], and confirmed that *K. marxianus* can be regarded as a good secretor, acting as a thermally flexible cell factory able to secrete active forms of an homologous enzyme in a wide range of temperatures.

### Secretion of heterologous proteins

In a previous study the comparison of expression and secretion of recombinant proteins at different temperatures has been carried out by using different hosts [28]. Herein, *K. marxianus* L3 was challenged for the secretion of three recombinant proteins in a wide range of temperatures. Specific growth rates (μ) were determined for each recombinant strain expressing the heterologous proteins HSA, GAA, or KlSod1p at 5, 15, 30, 40, and 45°C (Table 1). The recombinant strains were not able to grow at 45°C, possibly because of a low segregational stability of recombinant plasmids at the highest growth temperature, as reported in both prokaryotic and eukaryotic hosts [29,30].

In the explored range of temperature, the μ increased by raising the temperature, with the exception of HSA that presented μ_40°C_ < μ_30°C_. When heterologous proteins were expressed, generally μ were lower respect to the host (Table 1), reflecting a higher metabolic load [31,32].

### HSA production

**Figure 2:**
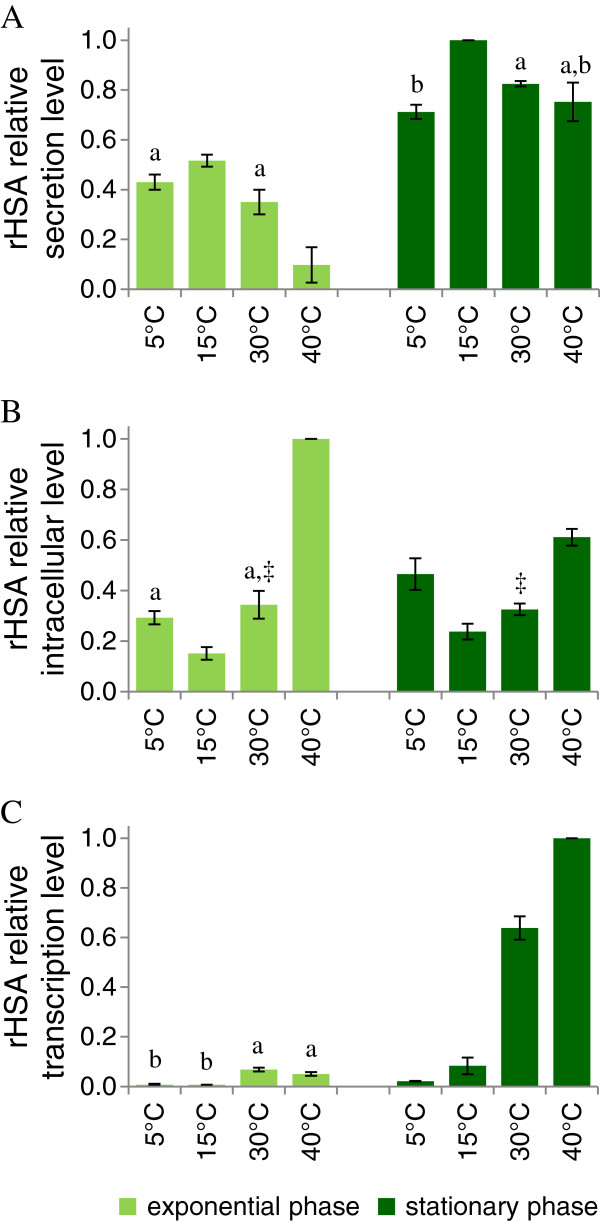
**Effects of the growth temperature on HSA secretion.** Culture samples, grown at 5 - 40°C, have been analyzed both at the growth and stationary phases. **A**) Western blot analysis of secreted rHSA; **B**) Western blot analysis of intracellular rHSA; **C**) Q-PCR analysis of rHSA mRNA. Data are reported as a function of growth phase and temperature. The values, means of three independent experiments ± standard deviations, have been normalized setting at 1 the highest value. Superscripts indicate statistically similar means (P > 0.05). Symbols are used to compare sample grown at the same temperature, collected at different growth phases (exponential or stationary). Letters are used to compare samples collected at the same growth phase, cultured at diverse temperatures.

HSA was successfully produced and secreted in the range between 5 and 40°C, and the growth phase affected specific HSA production (Figure 2A). The yields, expressed as relative abundances, showed a peak at 15°C both in exponential and stationary phases. A notably secretion efficiency was observed at all tested temperatures, when compared to the highest reference value (100% at 15°C). Compared to secretion levels, intracellular HSA production in both the exponential and stationary phases presented the opposite behavior, being the lowest at 15°C and the highest at 40°C (Figure 2B). Conceivably, the lower intracellular HSA at 15°C was associated to a more efficient secretion, and *vice versa* for the higher temperatures. A RT-PCR analysis was performed to determine transcription levels at the different temperatures (Figure 2C). At 5°C and 15°C very low amounts of HSA mRNA were observed during both exponential and stationary phases. At the higher temperatures, the relative amount of the messenger was always greater in stationary phase, and increased by rising the temperature. Although high temperatures seem not to drive the *ADH4* promoter activity in *S. cerevisiae* or in *K. lactis*[33], we could not exclude this possibility in the *K. marxianus* background. This aspect will deserve further investigations.

### GAA production

**Figure 3:**
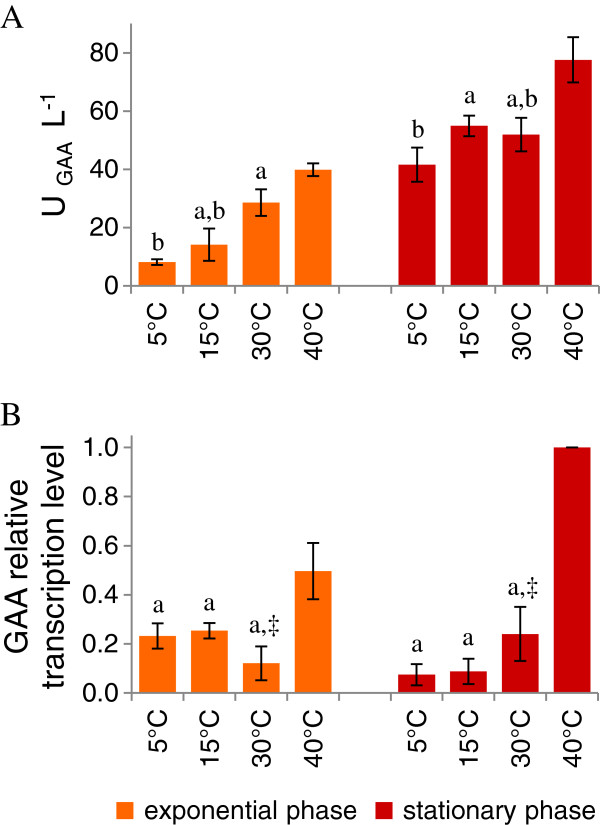
**Effects of the growth temperature on GAA secretion.** Culture samples, grown at 5 - 40°C, have been analyzed both at the growth and stationary phases. **A**) extracellular GAA activity; **B**) northern blot analysis of GAA mRNA. Data are means of three independent experiments ± standard deviations. The values, means of three independent experiments ± standard deviations, have been normalized setting at 1 the highest value. Superscripts indicate statistically similar means (P > 0.05). Symbols are used to compare samples grown at the same temperature, collected at different phases (exponential or stationary). Letters are used to compare samples collected at the same growth phase, cultured at different temperatures.

Heterologous GAA was successfully produced by *K. marxianus* at all the tested temperatures and the active enzyme accumulated in the medium over the time (Figure 3A). During the exponential phase production increased by rising the growth temperature up to 40°C. In the stationary phase, the total amount of active GAA secreted in the medium was similar over the range of temperatures between 5 and 30°C, whereas it was higher at 40°C. Transcript analysis showed that the relative amount of GAA mRNA had a slight variation between 5°C and 30°C in both growth phases; a significant increase was instead observed at 40°C (p < 0.05) (Figure 3B).

### KlSod1p production

**Figure 4:**
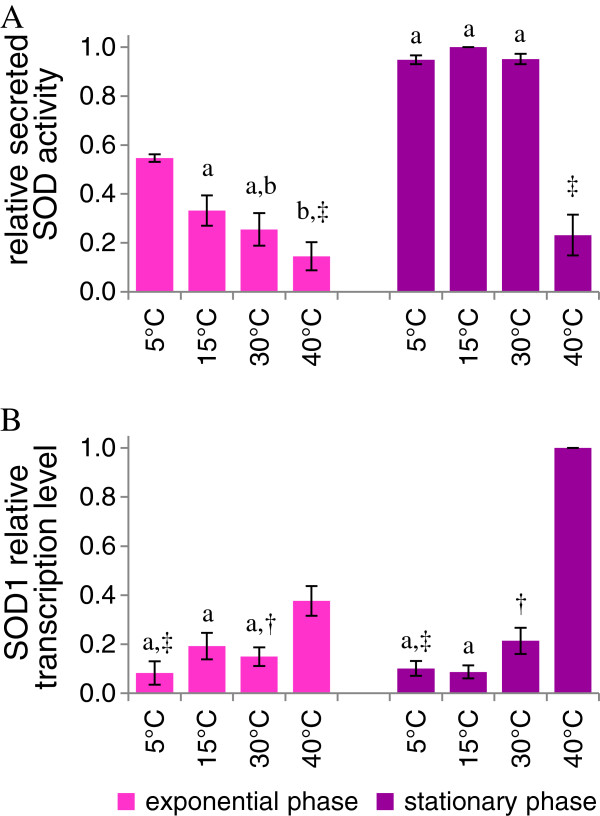
**Effects of the growth temperature on Sodp secretion.** Culture samples, grown at 5 - 40°C, have been analyzed both at the growth and stationary phases. **A**) extracellular SOD activity measured by zymogram analysis; **B**) northern blot analysis of SOD1 mRNA. Data are means of three independent experiments ± standard deviations. The values, means of three independent experiments ± standard deviations, have been normalized setting at 1 the highest value. Superscripts indicate statistically similar means (P > 0.05). Symbols are used to compare sample grown at the same temperature, collected at different growth phases (exponential or stationary). Letters are used to compare samples collected in the same growth phase, cultured at different temperatures.

The secretion efficiency of active Sod1p was compared at different temperatures by quantitative analysis of zymogram bands (Figure 4A). During the exponential phase, the capability to secrete active Sod1p increased by lowering the temperature. In stationary phase the production of active enzyme was similar between 5°C and 30°C and was significantly reduced at 40°C.

Transcriptional analysis showed that *SOD1* expression was almost not responsive to temperature up to 30°C; the level of transcript increased significantly at 40°C (Figure 4B). Actually, the genes *KmSOD1* of *K. marxianus* and *KlSOD1* of *K. lactis* present a 83% identity, it is then conceivable that the employed probe hybridized to both transcripts. Furthermore, endogenous Sod1p should be upregulated as a key part of a heat shock response. In *S. cerevisiae* oxidative stress and antioxidant enzymes play a pivotal role in heat-induced cell death and it has been observed that overexpression of catalase and superoxide dismutase genes caused increased thermotolerance [34].

Taken together, our results support *K. marxianus* L3 as a thermally flexible cell factory. This strain can be adopted in a process optimized for the ‘most productive temperature’ whenever no particular constraints were placed by the produced protein. Conversely, the strain L3 can be employed at the ‘most suitable temperature’ in case of specific protein requirements (*e.g.* stability at low temperature).

## Conclusions

In this study, the yeast *K. marxianus* has proved to be a handy model system to investigate the impact of temperature on cell factory efficiency. It resulted in a thermally flexible host able to produce and secrete three different recombinant proteins between 5 and 40°C. Notably, for the first time, the impact of a wide range of temperatures on the production of diverse heterologous proteins has been explored by using a single biotechnological platform. A peculiar behavior of each recombinant protein with respect to the temperature was observed, strengthening the advantage of using *K. marxianus* as unique host. Because of its ability to grow between 5°C and 40°C, and efficiently secrete heterologous proteins, it can be exploited as a first-strike model system to study the temperature tuning and optimization of the production. Moreover *K. marxianus* meets the criteria of process safety and the demand for fast growth and high yield biomass.

## Methods

### Strains and media

*Kluyveromyces marxianus* L3 [35] was used as expression host. Yeast cells were grown in YPD medium (10 g l^−1^yeast extract, 10 g l^−1^ peptone, and 20 g l^−1^glucose). Supplement of antibiotic G418 (0.2 g l^−1^) was added for the maintenance of recombinant plasmids. Standard batch cultures were carried out in 500 ml baffled Erlenmeyer flasks with 50 ml YPD medium. Flasks were inoculated (10% v/v) with exponential phase cultures, grown at the same temperature, to an OD_600_ of 1.0, then were incubated at 5, 15, 30, 40, and 45°C for 7, 4, 3, 2 and 2 days, respectively. For each cultures, samples corresponding to the exponential growth phase and to the stationary phase were analyzed. For strains harboring the plasmids pYG-Kl*KlSOD1*[22] and pGAM-GAM [20], heterologous protein expression was induced after 24 h of growth by the addition of ethanol to a final concentration of 2% v/v. All the experiments were performed in triplicate.

*Escherichia coli* DH5α (φ80*lacZ*Δ*M15*, *recA1, end A1, gyr A96, thi-1, hsd R17, rel A1*) was used for general cloning purposes.

### Plasmids and DNA manipulation

The pGM-GAM, pYG132, and pYG-K1*KlSOD1* plasmids were utilized for the expression of the reporter proteins. pGM-GAA contains the glucoamylase (GAA) gene from the yeast *Arxula adeninivorans* under the control of the *S. cerevisiae GAPDH* promoter and of the *S. cerevisiae PHO5* terminator [20]. pYG132 harbors an expression cassette for the secretion of the recombinant human serum albumin (HSA), driven by the native signal sequence, under the control of the ethanol inducible *K. lactis ADH4* promoter and of the *K. lactis PGK* terminator [21]. pYG-K1*KlSOD1* carries the *K. lactis KlSOD1* gene encoding the the Cu^2+^/Zn^2+^ superoxide dismutase fused with the K1 secretion signal, under the control of the *K. lactis ADH4* promoter and *PGK* terminator [22]. *K. marxianus* L3 was transformed by electroporation with a Biorad Gene-Pulser apparatus, as reported in Raimondi et al. [22].

### Calculation of the specific growth rate, biomass yield and volumetric productivity

For biomass determination, an appropriate volume of the culture was centrifuged. The pellet was washed in d.d. water and was desiccated under an infrared heat lamp. The dry weight was determined gravimetrically and the biomass yield was expressed as grams of dry weight *per* litre of culture broth. The specific growth rate (μ) was calculated using dry weight values in the exponential phase of the growth curve. Glucose consumption was analyzed by an HPLC equipped with refractive index detector (HPLC System, 1200 Series, Agilent Technologies, Santa Clara, CA). The analysis was performed with an Aminex HPX-87H ion exclusion column and 0.005 M H_2_SO_4_ (0.6 ml min^-1^) as the mobile phase. The volumetric productivities of biomass Q_X_ were calculated by dividing the biomass yield with the corresponding culture time.

### β-fructofuronidase activity

β-fructofuronidase was determined as inulinase activity according to Burkert et al. [36]. Culture was centrifuged for 5 minutes at 18000 g and 1 ml of supernatant, properly diluted in acetate buffer 0.1 M at pH 5, was mixed with 9 ml of 20 g l^-1^ sucrose dissolved in acetate buffer. The sample was incubated at 60°C and the rate of glucose and fructose production was determined by the 3,5-dinitrosalicylic acid method [37]. One unit of invertase is defined as the amount of enzyme catalyzing the liberation of 1 μmol of reducing sugars per minute under the conditions above mentioned.

### Western-blot analysis of recombinant HSA

For HSA Western-blot analysis, proteins from the supernatant (10 μl of 1:10 dilution) were separated with SDS-PAGE using the buffer system of Laemmli [38] and 12% acrylamide gels. After electroblotting onto a polyvinylidene difluoride membrane (Biorad), target proteins were detected with specific polyclonal antibodies of rabbit. Anti-HSA primary antibodies (Sigma) were used in 1:10,000 dilution. Monoclonal anti-rabbit IgG conjugated with peroxidase (Promega) were used as secondary antibodies. Immunologically active proteins were visualized with enhanced chemiluminescence detection system (GE Healthcare), according to the manufacturer’s instructions. The densitometric analysis was performed with an image analyzer (Phoretix 1D; Non Linear Dynamics Ltd.).

### RT-PCR analysis

Total RNA of *K. marxianus* cells was extracted by the hot phenol method [39]. RNA was quantified by absorbance (A_260_) and subjected to TURBO DNase treatment according to the manufacturer’s instructions (Ambion). Reverse transcription was performed using a Promega Reverse Transcription System with 1 μg total RNA to yield 20 μl cDNA. After cDNA generation, samples were purified, quantified with Nanodrop™ 3300 (Thermo Scientific), diluted with DEPC water (Ambion) to a final concentration of 10 ng μl^-1^, and 2 μl were used for SYBR Green SensiMix (Bioline). Oligonucleotides (purchased from SIGMA) were designed with Primer3 (http://frodo.wi.mit.edu/primer3/ website), considering an amplicon size of 95–220 bp and a Tm of approximately 55°C. For the HSA the primers used were 5^′^- GTTGCAACTCTTCGTGAAAC-3^′^ and 5^′^- AAGTAAGGATGTCTTCTGGC-3^′^; for the *KmACT1* they were 5^′^- CTCCTTGCCTCATGCTATC-3^′^ and 5^′^-GAAGGAGTAACCACGTTCAG-3^′^.

### Glucoamilase activity

GAA activity was determined at 37°C as starch-hydrolyzing activity of the cell-free culture broths, according to Morlino et al. [20] One unit of GAA activity was defined as the quantity of enzyme needed to decrease the absorbance at 580 nm by one absorbance unit per minute. GAA activity was expressed as volumetric activity (U l^-1^).

### Superoxide dismutase activity

SOD activity was determined by on native gel by the nitroblue tetrazolium method [40]. During illumination with light, the gel became uniformly blue-purple except at positions containing active SOD protein. The densitometric analysis was carried out with the image analyzer Phoretix 1D (Non Linear Dynamics Ltd).

### Northern blot analysis

Total RNA was prepared by extraction with hot acidic phenol [41]. Northern blot analysis was performed as previously described according to Sambrook et al. [42]. The *A. adeninivorans* GAA probe corresponded to the 2.1-kb *Hin*dIII region of the pGM-GAM plasmid. The 0.7 kb fragment of the Kl*KlSOD1* gene, amplified using the set of primers K1-KlSOD1_f (5^′^-GC AAG CTT ATG AAT ATA TTT TAC ATA TTT TTG TTT TTG CTG TCA TTC GTT CAA GGT ACC CGG GGA GTT AAT GCA GTT GCA G-3^′^) and KlSOD1_r (5^′^-GC AAG CTT TTA AGC GTT AGA GAT ACC-3^′^), was used to detect *KlSOD1* mRNA. The probes were labeled with [α-^32^P]dATP by use of the Ready Prime DNA labeling system (GE Healthcare), according to the manufacturer’s instructions.

### Statistical analysis

All values are means of three separate experiments. Differences in means were analyzed using Student’s *t* test with independent measures. Differences were considered statistically significant if P < 0.05.

## Competing interests

The authors declare that they have no competing interests.

## Authors’ contributions

SR, DU and MR conceived the study, participated in its design and wrote the manuscript. SR, EZ, and AA performed the experimental work and drafted the manuscript. CP has been involved in drafting the manuscript and revising it critically. All authors read and approved the final manuscript.

## References

[B1] Iborra F High efficiency transformation of *Kluyveromyces marxianus* by a replicative plasmid Curr Genet 1993 24 181 183 10.1007/BF00324685 8358827

[B2] Fonseca GG Heinzle E Wittmann C Gombert AK The yeast *Kluyveromyces marxianus* and its biotechnological potential Appl Microbiol Biotechnol 2008 79 339 354 10.1007/s00253-008-1458-6 18427804

[B3] Diniz RH Silveira WB Fietto LG Passos FM The high fermentative metabolism of *Kluyveromyces marxianus* UFV-3 relies on the increased expression of key lactose metabolic enzymes Antonie Van Leeuwenhoek 2012 101 541 550 10.1007/s10482-011-9668-9 22068918

[B4] Dos Santos VC Bragança CR Passos FJ Passos FM Kinetics of growth and ethanol formation from a mix of glucose/xylose substrate by *Kluyveromyces marxianus* UFV-3 Antonie Van Leeuwenhoek 2013 103 153 161 10.1007/s10482-012-9794-z 22965752

[B5] López-Alvarez A Díaz-Pérez AL Sosa-Aguirre C Macías-Rodríguez L Campos-García J Ethanol yield and volatile compound content in fermentation of agave must by *Kluyveromyces marxianus* UMPe-1 comparing with *Saccharomyces cerevisiae* baker’s yeast used in tequila production J Biosci Bioeng 2012 113 5 614 618 10.1016/j.jbiosc.2011.12.015 22280963

[B6] Lee KS Kim JS Heo P Yang TJ Sung YJ Cheon Y Koo HM Yu BJ Seo JH Jin YS Park JC Kweon DH Characterization of *Saccharomyces cerevisiae* promoters for heterologous gene expression in *Kluyveromyces marxianus* Appl Microbiol Biotechnol 2012 Epub ahead of print 10.1007/s00253-012-4306-7 22911091

[B7] Jeong H Lee DH Kim SH Kim HJ Lee K Song JY Kim BK Sung BH Park JC Sohn JH Koo HM Kim JF Genome sequence of the thermotolerant yeast *Kluyveromyces marxianus var. marxianus* KCTC 17555 Eukaryot Cell 2012 11 12 1584 1585 10.1128/EC.00260-12 23193140 PMC3536290

[B8] Rocha SN Abrahão-Neto J Cerdán ME Gombert AK González-Siso MI Heterologous expression of a thermophilic esterase in Kluyveromyces yeasts Appl Microbiol Biotechnol 2011 89 375 385 10.1007/s00253-010-2869-8 20862582

[B9] Rocha SN Abrahão-Neto J Cerdán ME González-Siso MI Gombert AK Heterologous expression of glucose oxidase in the yeast *Kluyveromyces marxianus* Microb Cell Fact 2010 9 4 10.1186/1475-2859-9-4 20092622 PMC2817671

[B10] Raimondi S Uccelletti D Amaretti A Leonardi A Palleschi C Rossi M Secretion of *Kluyveromyces lactis* Cu/Zn SOD: strategies for enhanced production Appl Microbiol Biotechnol 2010 86 871 878 10.1007/s00253-009-2353-5 20012282

[B11] Foukis A Stergiou PY Theodorou LG Papagianni M Papamichael EM Purification, kinetic characterization and properties of a novel thermo-tolerant extracellular protease from *Kluyveromyces marxianus* IFO 0288 with potential biotechnological interest Bioresour Technol 2012 123 214 220 22940322 10.1016/j.biortech.2012.06.090

[B12] Rossi M Buzzini P Cordisco L Amaretti A Sala M Raimondi S Ponzoni C Pagnoni UM Matteuzzi D Growth, lipid accumulation, and fatty acid composition in obligate psychrophilic, facultative psychrophilic, and mesophilic yeasts FEMS Microbiol Ecol 2009 69 363 372 10.1111/j.1574-6941.2009.00727.x 19624740

[B13] García-Fruitós E Vazquez E Gonzalez-Montalbán N Ferrer-Miralles N Villaverde A Analytical approaches for assessing aggregation of protein biopharmaceuticals Curr Pharm Biotechnol 2011 12 1530 1536 10.2174/138920111798357339 21542795

[B14] Maldonado LMTP Hernández VEB Rivero EM de la Rosa AP B Flores JLF Acevedo LGO De León Rodríguez A Optimization of culture conditions for a synthetic gene expression in *Escherichia coli* using response surface methodology: the case of human interferon beta Biomol Eng 2007 24 217 222 10.1016/j.bioeng.2006.10.001 17126075

[B15] Noguère C Larsson AM Guyot JC Bignon C Fractional factorial approach combining 4 *Escherichia coli* strains, 3 culture media, 3 expression temperatures and 5 N-terminal fusion tags for screening the soluble expression of recombinant proteins Protein Expr Purif 2012 84 204 213 10.1016/j.pep.2012.05.011 22705765

[B16] Baneyx F Mujacic M Recombinant protein folding and misfolding in *Escherichia coli* Nat Biotechnol 2004 22 1399 1408 10.1038/nbt1029 15529165

[B17] Dragosits M Stadlmann J Albiol J Baumann K Maurer M Gasser B Sauer M Altmann F Ferrer P Mattanovich D The effect of temperature on the proteome of recombinant *Pichia pastoris* J Proteome Res 2009 8 1380 1392 10.1021/pr8007623 19216534

[B18] Li Z Xiong F Lin Q D’Anjou M Daugulis AJ Yang DS Hew CL Low-temperature increases the yield of biologically active herring antifreeze protein in *Pichia pastoris* Protein Expr Purif 2001 21 438 445 10.1006/prep.2001.1395 11281719

[B19] Gasser B Maurer M Rautio J Sauer M Bhattacharyya A Saloheimo M Penttilä M Mattanovich D Monitoring of transcriptional regulation in *Pichia pastoris* under protein production conditions BMC Genomics 2007 8 179 10.1186/1471-2164-8-179 17578563 PMC1919374

[B20] Morlino GB Tizzani L Bianchi MM Frontali L Inducible amplification of gene copy number and heterologous proten production in the yeast *Kluyveromyces lactis* Appl Environ Microbiol 1999 65 4808 4813 10543790 10.1128/aem.65.11.4808-4813.1999 PMC91648

[B21] Saliola M Mazzoni C Solimando N Crisà A Falcone C Jung G FleerR: **Use of the *****KlADH4 *****promoter for ethanol-dependent production of recombinant Human Serum Albumin in *****Kluyveromyces lactis*** Appl Environ Microbiol 1999 65 53 60 9872759 10.1128/aem.65.1.53-60.1999 PMC90982

[B22] Raimondi S Uccelletti D Matteuzzi D Pagnoni UM Rossi M Palleschi C Characterization of the superoxide dismutase *SOD1* gene of *Kluyveromyces marxianus* L3 and improved production of SOD activity Appl Microbiol Biotechnol 2008 77 1269 1277 10.1007/s00253-007-1270-8 18040680

[B23] Lane MM Burke N Karreman R Wolfe KH O’Byrne CP Morrissey JP Physiological and metabolic diversity in the yeast *Kluyveromyces marxianus* Antonie Van Leeuwenhoek 2011 100 507 519 10.1007/s10482-011-9606-x 21674230

[B24] Fonseca GG Gombert AK Heinzle E Wittmann C Physiology of the yeast *Kluyveromyces marxianus* during batch and chemostat cultures with glucose as the sole carbon source FEMS Yeast Res 2007 7 422 435 10.1111/j.1567-1364.2006.00192.x 17233766

[B25] Masoud W Jespersen L Pectin degrading enzymes in yeasts involved in fermentation of Coffea arabica in East Africa Int J Food Microbiol 2006 110 291 296 10.1016/j.ijfoodmicro.2006.04.030 16784790

[B26] Arrizon J Morel S Gschaedler A Monsan P Purification and substrate specificities of a fructanase from *Kluyveromyces marxianus* isolated from the fermentation process of Mezcal Bioresour Technol 2011 102 3298 3303 10.1016/j.biortech.2010.10.071 21067917

[B27] Rouwenhorst RJ Ritmeester WS Scheffers WA Van Dijken JP Localization of inulinase and invertase in *Kluyveromyces* species Appl Environ Microbiol 1990 56 3329 3336 2268150 10.1128/aem.56.11.3329-3336.1990 PMC184950

[B28] Dragosits M Frascotti G Bernard-Granger L Vázquez F Giuliani M Baumann K Rodríguez-Carmona E Tokkanen J Parrilli E Wiebe MG Kunert R Maurer M Gasser B Sauer M Branduardi P Pakula T Saloheimo M Penttilä M Ferrer P Luisa Tutino M Villaverde A Porro D Mattanovich D Influence of growth temperature on the production of antibody Fab fragments in different microbes: a host comparative analysis Biotechnol Prog 2011 27 38 46 10.1002/btpr.524 21312353

[B29] Zhang Y Li T Liu J Low temperature and glucose enhanced T7 RNA polymerase-based plasmid stability for increasing expression of glucagons-like peptide-2 in *Escherichia coli* Protein Expr Purif 2003 29 132 139 10.1016/S1046-5928(03)00002-0 12729734

[B30] Zhang Z Moo-Young M Chisti Y Plasmid stability in recombinant *Saccharomyces cerevisiae* Biotechnol Adv 1996 14 401 435 10.1016/S0734-9750(96)00033-X 14540156

[B31] Glick BR Metabolic load and heterologous gene expression Biotechnol Adv 1995 13 247 261 10.1016/0734-9750(95)00004-A 14537822

[B32] Neubauer P Lin HY Mathiszik B Metabolic load of recombinant protein production: inhibition of cellular capacities for glucose uptake and respiration after induction of a heterologous gene in *Escherichia coli* Biotechnol Bioeng 2003 83 53 64 10.1002/bit.10645 12740933

[B33] Mazzoni C Santori F Saliola M Falcone C Molecular analysis of UAS(E), a *cis* element containing stress response elements responsible for ethanol induction of the *KlADH4* gene of *Kluyveromyces lactis* Res Microbiol 2000 151 19 28 10.1016/S0923-2508(00)00131-5 10724480

[B34] Davidson JF Whyte B Bissinger PH Schiestl RH Oxidative stress is involved in heat-induced cell death in *Saccharomyces cerevisiae* PNAS 1996 93 5116 5121 10.1073/pnas.93.10.5116 8643537 PMC39416

[B35] Dellomonaco C Amaretti A Zanoni S Pompei A Matteuzzi D Rossi M Fermentative production of superoxide dismutase with *Kluyveromyces marxianus* J Ind Microbiol Biotechnol 2007 34 27 34 16909269 10.1007/s10295-006-0158-4

[B36] Burkert JFM Kalil SJ Maugeri Filho F Rodrigues MI Parameters optimization for enzymatic assays using experimental design Braz J ChemEng 2006 23 163 170

[B37] Miller GL Use of dinitrosalisylic acid reagent for determination of reducing sugar Anal Chem 1959 31 426 428 10.1021/ac60147a030

[B38] Laemmli UK Cleavage of Structural Proteins during the Assembly of the Head of Bacteriophage T4 Nature 1970 227 680 685 10.1038/227680a0 5432063

[B39] Schmitt ME Brown TA Trumpower BL A rapid and simple method for preparation of RNA from *Saccharomyces cerevisiae* Nucleic Acids Res 1990 18 3091 3092 10.1093/nar/18.10.3091 2190191 PMC330876

[B40] McCord JM Keele BB Fridovich I An enzyme-based theory of obligate anaerobiosis: the physiological function of superoxide dismutase Proc Natl Acad Sci 1971 68 1024 1027 10.1073/pnas.68.5.1024 4995818 PMC389105

[B41] Ausubel FM Brent R Kingston RE Moore DD Seidman JG Smith JA Struhl K Current protocols in molecular biology 1994 New York: Greene Publishing Associates and Wiley-Interscience

[B42] Sambrook J Fritsch EF Maniatis T Molecular cloning: a laboratory manual 2001 2 New York, NY: Cold Spring Harbor Laboratory Press

